# Machine learning to improve frequent emergency department use prediction: a retrospective cohort study

**DOI:** 10.1038/s41598-023-27568-6

**Published:** 2023-02-03

**Authors:** Yohann M. Chiu, Josiane Courteau, Isabelle Dufour, Alain Vanasse, Catherine Hudon

**Affiliations:** 1 Faculté de Pharmacie, Université Laval, Québec, QC Canada; 2 Département de Médecine de Famille et de Médecine d’urgence, Faculté de Médecine et des Sciences de la Santé, Université de Sherbrooke, Sherbrooke, QC Canada; 3 Centre de Recherche du Centre Hospitalier Universitaire de Sherbrooke, Université de Sherbrooke, Sherbrooke, QC Canada; 4 Département d’épidémiologie, Biostatistique et Santé au Travail, Faculté de Médecine, Université McGill, Montréal, QC Canada

**Keywords:** Health services, Public health, Epidemiology

## Abstract

Frequent emergency department use is associated with many adverse events, such as increased risk for hospitalization and mortality. Frequent users have complex needs and associated factors are commonly evaluated using logistic regression. However, other machine learning models, especially those exploiting the potential of large databases, have been less explored. This study aims at comparing the performance of logistic regression to four machine learning models for predicting frequent emergency department use in an adult population with chronic diseases, in the province of Quebec (Canada). This is a retrospective population-based study using medical and administrative databases from the *Régie de l’assurance maladie du Québec*. Two definitions were used for frequent emergency department use (outcome to predict): having at least three and five visits during a year period. Independent variables included sociodemographic characteristics, healthcare service use, and chronic diseases. We compared the performance of logistic regression with gradient boosting machine, naïve Bayes, neural networks, and random forests (binary and continuous outcome) using Area under the ROC curve, sensibility, specificity, positive predictive value, and negative predictive value. Out of 451,775 ED users, 43,151 (9.5%) and 13,676 (3.0%) were frequent users with at least three and five visits per year, respectively. Random forests with a binary outcome had the lowest performances (ROC curve: 53.8 [95% confidence interval 53.5–54.0] and 51.4 [95% confidence interval 51.1–51.8] for frequent users 3 and 5, respectively) while the other models had superior and overall similar performance. The most important variable in prediction was the number of emergency department visits in the previous year. No model outperformed the others. Innovations in algorithms may slightly refine current predictions, but access to other variables may be more helpful in the case of frequent emergency department use prediction.

## Introduction

Although definitions may vary, individuals who visit emergency department (ED) at least three times per year are considered as “frequent users”^1–3^. Frequent ED users often display heterogeneous profiles—a combination of mental health disorders, physical comorbidities, and low socioeconomic status^1,4,5^—leading to complex needs that are not adequately dealt with in an ED^6^. A significant proportion of frequent ED users have numerous chronic diseases, such as coronary artery disease or chronic obstructive pulmonary disease^4,7^. Those conditions could be managed in primary care, preventing acute deteriorations that lead to ED use^8^. Since frequent ED use for complex needs may occur because those needs have not been adequately addressed in a primary care context, this type of ED use is considered suboptimal. As an indicator of unmet needs, it is associated with negative outcomes for patients (e.g., higher hospital admissions or mortality rates^9,10^). Furthermore, ED costs are generally higher than those in a primary care setting, resulting in a socioeconomical burden for the health system^7,11,12^. In the province of Quebec (Canada), frequent ED users with chronic diseases represent 9.2% of all the ED users but account for 28.8% of all ED visits^13^. Furthermore, a recent Canadian census shows that chronic condition prevalence will increase as the population age; the burden on the healthcare system is then likely to increase^14^.

Targeted interventions such as case management have been shown to help reduce ED visits and ED costs, while improving patient satisfaction and clinical outcomes^2,15,16^. In this context, being able to accurately predict frequent ED use is relevant to target users who may really benefit from it. Much work has been done with statistical models in this direction. In particular, logistic regression (LR) is a standard and widely used statistical model^17^. However, with the constant improvement of quantity and quality of measurements (electronic health records), statistical models, and computer capacity, modern machine learning (ML) models are becoming more and more popular. Previous studies have predicted frequent ED use for a specific issue^18,19^ or in a local hospital^20^ successfully using ML models other than LR. Yet, no study has been conducted comparing predictive power of ML models in a general population and considering chronic diseases.

This study aims at comparing the performance of a logistic regression to four ML models for frequent emergency department use in an adult population with chronic diseases, in the province of Quebec (Canada).

## Methods

All methods in this study were carried out in accordance with the TRIPOD guidelines for model development and validation (see the Supplementary material Table S1)^21^.

### Study design and data sources

This is a population-based retrospective cohort study. We used medico-administrative databases from the health insurance board of the province of Quebec (*Régie de l’assurance maladie du Québec*), which manages health insurance plan for Quebec citizens. The following files were used:

1. The patient demographic register, which contains information about the sex, date of birth, date of death (if applicable), and place of residence of the patient;
2. The physician reimbursement claim register, which contains information about medical services provided by a fee-for-service physician in Quebec: date of service, place of service (emergency, medical clinic, etc.), physician specialty, diagnosis (International Classification of Diseases, ninth revision or ICD-9), and the medical act procedure performed by the physician;
3. The hospital register, which contains information about the reasons for hospitalization (main diagnosis and up to 25 secondary diagnoses coded in ICD-10), dates of admission and release from hospital, and all medical procedures performed during the hospitalization.

### Selection of participants

The study population included all adults (18 years and older) living in the province of Quebec, with at least one ED visit during the inclusion period, i.e., between the 1st of January 2012 and the 31st of December 2013, diagnosed with at least one chronic condition, and without dementia. Patients with dementia may have special needs compared to cognitively intact patients and were thus not included. In this study, the diseases considered were those from the Canadian Institute for Health Information (see the Supplementary material Table S2): asthma, chronic obstructive pulmonary disease (COPD), congestive heart failure (CHF), coronary artery disease (CAD), diabetes, epilepsy, and high blood pressure (HBP)^22^. Those specific conditions, also known as ambulatory care sensitive conditions, are a set of chronic diseases for which timely intervention in primary care could reduce the risk of hospitalization or the occurrence of acute episodes for those diseases^23–25^. The index date was randomly assigned as one ED visit among all ED visits occurring during the inclusion period^26^. The index date is then used as a “starting point” for measuring patient characteristics, such as ED use, age, or diagnoses.

**Figure 1:**
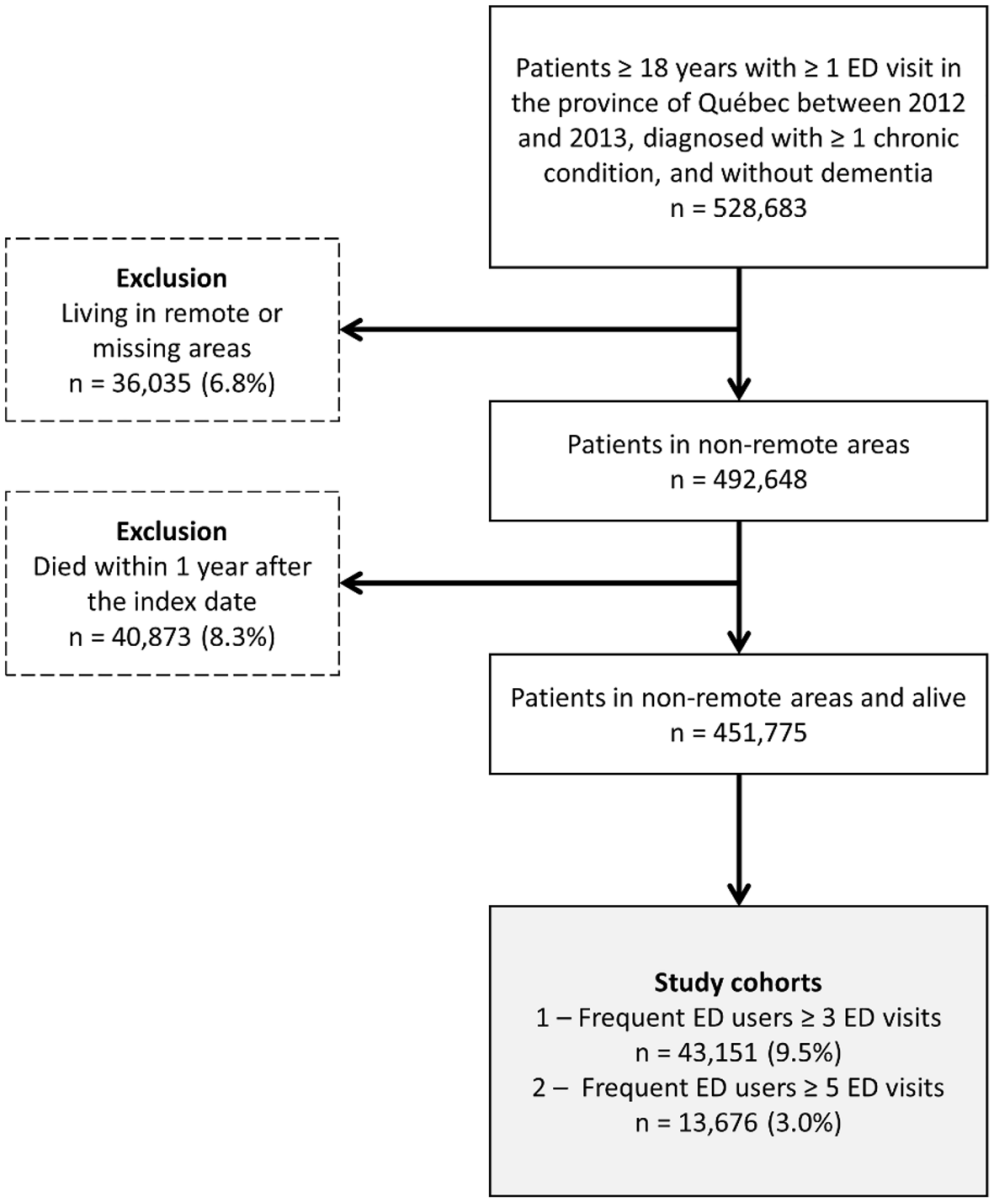
Flowchart of the study cohorts.

There were two exclusion criteria (Fig. 1). First, patients living in remote areas were excluded (6.8%). Remote areas were defined as municipalities with fewer than 10,000 inhabitants with weak or no metropolitan influence zone (the percentage of resident employed labour force who commute to work in urban areas is less than 5%). This exclusion ensured that remote residents who tend to use ED as an alternative to walk-in clinics (as there are fewer primary care alternatives^27,28^) were not included. However, patients living in municipalities with fewer than 10,000 inhabitants with high or moderate metropolitan influence were included. Secondly, patients who died during the year after their index date (8.3%) were excluded as they can require specialized healthcare, such as patients at the end of life^29,30^. Besides, that exclusion helped reducing immortal time bias^31^.

### Outcome and independent variables

Frequent ED use was investigated using two different definitions: (1) having at least three visits (“frequent users 3”) and (2) having at least five visits (“frequent users 5”) during the year following the index date (as mentioned in the previous subsection, the index date is an assigned ED visit between 2012 and 2013). Those definitions were chosen amongst the most common ones in order to compare performance in two populations that were different, yet still considered frequent users.

Independent variables (or predictors) considered at the index date were sex, age, residential area (metropolitan: ≥ 100,000; small town: 10,000–100,000; rural: < 10,000 with high or moderate metropolitan influence), material and social deprivation indices^32^, public prescription drug insurance plan status (PPDIP, see below for the different statuses), having been hospitalized in the two years before the index date, the number of previous ED visits during the year before the index date (PV), and the combined comorbidity index of Charlson (CCI^33^). The following diagnoses were considered: chronic disease (one diagnosis for each condition, i.e. asthma, COPD, CHF, CAD, diabetes, epilepsy, and HBP), chronic non-cancer pain (CNCP)^34^, injury, common mental disorders (CMD)^35^, serious mental disorders (SMD)^35^, alcohol abuse, and drug abuse. Each condition was identified using the reported diagnoses in the hospital register (one diagnosis) or in the physician reimbursement claim register (at least two diagnoses), during a two-year period before the index date.

Regarding PPDIP status, the Quebec province has four different statuses: “regular recipient of PPDIP”, “admissible to PPDIP and age ≥ 65 years with guaranteed income supplement” (GIS), “not admissible to PPDIP” (individuals with a private insurance plan), or “admissible to PPDIP and being a recipient of last-resort financial assistance” (LRFA)^36^.

There were less than 5% missing observations, mainly for material and social deprivation indices, and those observations were kept.

### Statistical analysis

Frequent ED use prediction is a case of supervised learning, meaning that there are explicit labelled classes (i.e., frequent user or not). Along with logistic regression (LR), four ML predictive models amongst the most efficient for predicting a binary outcome^38^ were evaluated:

1. Gradient boosting machines (GBM) build an ensemble of successive decision trees; each tree is a weak learner that improves on the previous one using the residuals^39^. Tuning parameters were the learning rate and the trees depth.
2. Naïve Bayes (NB) model is based on Bayes’ theorem and uses a priori probabilities^40^. The tuning parameter was the Laplace smoothing for probabilities.
3. Neural networks (NN) feed data through interconnected hidden layers of “neurons”, which apply mathematical operations to the inputs (the independent variables)^41^. Tuning parameters were the number of neurons and the weight decay.
4. Random forests (RF) apply sequential splits to the data such that the separation is maximized in regards to a homogeneity criterion (i.e., the Gini index), resulting in a tree-like structure^40^. RF were evaluated with a binary (RF1) and a continuous outcome (RF2). Tuning parameters were the number of trees and the homogeneity criterion used.

The cohort was randomly divided in a training set (80% of the cohort) for building models and a testing set (remaining 20%) for evaluating performance^18,42^. This procedure is common in order to minimize overfitting, a sensitive issue when dealing with ML algorithms^43^. Area under the ROC curve (AUC), sensibility (SEN), specificity (SPE), positive predictive value (PPV), and negative predictive value (NPV) were computed to compare performances. AUC 95% confidence intervals were also computed using DeLong’s method^44^. The same reasoning was adopted as in Grinspan et al.^18^, the predictability of a model was judged on its AUC, based on 5 categories: poor (0.50–0.59), fair (0.60–0.69), good (0.70–0.79), very good (0.80–0.89) and excellent (0.90–1.0)^18^. The best cut-off thresholds were selected using Youden’s statistic ^45^ in order to compute sensitivity, specificity, positive predictive value, and negative predictive value. All tuning parameters were optimized by searching for the maximum AUC, but only the results with the selected parameters are presented here for clarity and brevity purposes.

Results from ML models (except LR) are not as directly interpretable as those from regression models, which straightforwardly assess the effect of predictive variables on the outcome with quantities such as odd ratios. However, ML framework allows for the evaluation of variable importance in a prediction model (also called feature importance). It was computed as the mean decrease in the Gini index in the case of GBM and RF, as the combinations of the absolute values of the weights for NN, and as the absolute value of the t-statistic for LR^43^. While it is not possible to compare variable importance directly from one model to another due to the models being different in nature, variable importance is still useful as an interpretable and relative quantity about the contribution of each predictor. In our models, all the variables are categorical and GBM, LR, and NN compute variable importance relative to a baseline category while RF computes an overall variable importance. Of note, there is no available variable importance measure when using the NB algorithm.

Sensitivity analyses were conducted on a population of frequent users with at least four visits and with a 50/50 training and testing sets.

Statistical significance level was set at α = 0.05 and differences in descriptive statistics were evaluated using chi-square tests. All analyses were performed with statistical software programs SAS (version 9.4) and R (version 4.2 with packages e1071, nnet, ranger, and xgboost).

### Ethics approval and consent to participate

The research ethics board of the *Centre intégré universitaire de santé et de services sociaux de l’Estrie – Centre hospitalier universitaire de Sherbrooke* (number MP-31–2017-1571 – Urgences-CPSA) approved this study. The need for informed consent was waived by the aforementioned research ethics board due to the retrospective nature of the study.

## Results

### Characteristics of participants

Out of 451,775 ED users, 43,151 (9.5%) and 13,676 (3.0%) were frequent users 3 and frequent users 5, respectively (Table 1). For both definitions, differences between frequent users and non-frequent users were statistically significant except for the residential area variable.

**Table 1:** Descriptive statistics for the different populations.

Variable	Total (%)	Non-frequent users 3 (%)	Frequent users 3 (%)	Non-frequent users 5 (%)	Frequent users 5 (%)
**Total**	451,775 (100)	408,624 (100)	43,151 (100)	438,099 (100)	13,676 (100)
**Female**	234,320 (51.9)	210,904 (51.6)	23,416 (54.3)	226,968 (51.8)	7352 (53.8)
**Age**					
18–34	23,723 (5.3)	21,179 (5.2)	2,544 (5.9)	22,775 (5.2)	948 (6.9)
35–54	83,393 (18.5)	76,291 (18.7)	7,102 (16.5)	80,977 (18.5)	2416 (17.7)
55–64	99,136 (21.9)	91,411 (22.4)	7,725 (17.9)	96,618 (22.1)	2518 (18.4)
65–74	116,323 (25.7)	105,977 (25.9)	10,346 (24.0)	113,198 (25.8)	3125 (22.9)
75–84	93,091 (20.6)	82,660 (20.2)	10,431 (24.2)	89,887 (20.5)	3204 (23.4)
≥ 85	36,109 (8.0)	31,106 (7.6)	5,003 (11.6)	34,644 (7.9)	1465 (10.7)
**PPDIP admissibility**					
Regular	170,044 (37.6)	155,489 (38.1)	14,555 (33.7)	165,854 (37.9)	4190 (30.6)
GIS	118,313 (26.2)	103,725 (25.4)	14,588 (33.8)	113,618 (25.9)	4695 (34.3)
Not admissible	129,608 (28.7)	121,314 (29.7)	8294 (19.2)	127,254 (29.0)	2354 (17.2)
LRFA	33,810 (7.5)	28,096 (6.9)	5714 (13.2)	31,373 (7.2)	2437 (17.8)
**Residential area**					
Metropolitan	302,097 (66.9)	274,825 (67.3)	27,272 (63.2)	293,601 (67.0)	8496 (62.1)
Small town	67,685 (15.0)	60,378 (14.8)	7307 (16.9)	65,331 (14.9)	2354 (17.2)
Rural	81,993 (18.1)	73,421 (18.0)	8572 (19.9)	79,167 (18.1)	2826 (20.7)
**Previous ED visits**					
≤ 1	354,465 (78.5)	334,708 (81.9)	19,757 (45.8)	350,197 (79.9)	4268 (31.2)
2–3	71,406 (15.8)	58,738 (14.4)	12,668 (29.4)	67,507 (15.4)	3899 (28.5)
4	11,282 (2.5)	7834 (1.9)	3448 (8.0)	9888 (2.3)	1394 (10.2)
5	5959 (1.3)	3653 (0.9)	2306 (5.3)	4926 (1.1)	1033 (7.6)
≥ 6	8663 (1.9)	3691 (0.9)	4972 (11.5)	5581 (1.3)	3082 (22.5)
**Previous hospitalization**	191,862 (42.5)	165,229 (40.4)	26,633 (61.7)	182,364 (41.6)	9498 (69.5)
**Material deprivation**					
Missing	15,928 (3.5)	13,976 (3.4)	1952 (4.5)	15,242 (3.5)	686 (5.0)
1	70,303 (15.6)	64,773 (15.9)	5530 (12.8)	68,731 (15.7)	1572 (11.5)
2	82,729 (18.3)	75,577 (18.5)	7152 (16.6)	80,578 (18.4)	2151 (15.7)
3	87,736 (19.4)	79,719 (19.5)	8017 (18.6)	85,238 (19.5)	2498 (18.8)
4	96,514 (21.4)	86,900 (21.3)	9614 (22.3)	93,399 (21.3)	3115 (22.8)
5	98,565 (21.8)	87,679 (21.5)	10,886 (25.2)	94,911 (21.7)	3654 (26.7)
**Social deprivation**					
Missing	15,928 (3.5)	13,976 (3.4)	1952 (4.5)	15,242 (3.5)	686 (5.0)
1	73,218 (16.2)	67,198 (16.4)	6020 (14.0)	71,435 (16.3)	1783 (13.0)
2	77,968 (17.3)	71,444 (17.5)	6524 (15.1)	75,997 (17.3)	1971 (14.4)
3	87,542 (19.4)	79,417 (19.4)	8125 (18.8)	85,127 (19.4)	2415 (17.7)
4	93,164 (20.6)	84,148 (20.6)	9016 (20.9)	90,239 (20.6)	2925 (21.4)
5	103,955 (23.0)	92,441 (22.6)	11,514 (26.7)	100,059 (22.8)	3896 (28.5)
**Comorbidity index**					
0	277,798 (61.5)	259,504 (63.5)	18,294 (42.4)	272,919 (62.3)	4879 (35.7)
1–2	98,228 (21.7)	86,976 (21.3)	11,252 (26.1)	94,558 (21.6)	3670 (26.8)
3–4	34,395 (7.6)	28,527 (7.0)	5868 (13.6)	32,248 (7.4)	2147 (15.7)
≥ 5	41,354 (9.2)	33,617 (8.2)	7737 (17.9)	38,374 (8.8)	2980 (21.8)
**Asthma**	47,514 (10.5)	41,438 (10.1)	6076 (14.1)	45,210 (10.3)	2304 (16.8)
**COPD**	62,975 (13.9)	51,842 (12.7)	11,133 (25.8)	58,694 (13.4)	4281 (31.3)
**CHF**	27,945 (6.2)	22,281 (5.5)	5664 (13.1)	25,793 (5.9)	2152 (15.7)
**CAD**	113,141 (25.0)	98,234 (24.0)	14,907 (34.5)	108,062 (24.7)	5079 (37.1)
**Diabetes**	151,951 (33.6)	135,269 (33.1)	16,682 (38.7)	146,325 (33.4)	5626 (41.1)
**Epilepsy**	11,538 (2.6)	9857 (2.4)	1681 (3.9)	10,864 (2.5)	674 (4.9)
**HBP**	245,449 (54.3)	220,607 (54.0)	24,842 (57.6)	237,388 (54.2)	8061 (58.9)
**Alcohol abuse**	10,678 (2.4)	8173 (2.0)	2505 (5.8)	9444 (2.2)	1234 (9.0)
**CNCP**	75,263 (16.7)	65,660 (16.1)	9603 (22.3)	71,859 (16.4)	3404 (24.9)
**CMD**	102,540 (22.7)	87,533 (21.4)	15,007 (34.8)	96,711 (22.1)	5829 (42.6)
**Drug abuse**	6908 (1.5)	4854 (1.2)	2054 (4.8)	5827 (1.3)	1081 (7.9)
**Injury**	160,577 (35.5)	140,479 (34.4)	20,098 (46.6)	153,400 (35)	7177 (52.5)
**SMD**	15,778 (3.5)	12,465 (3.1)	3313 (7.7)	14,281 (3.3)	1497 (10.9)

Percentages in brackets are relative to the column.

*PPDIP* public prescription drug insurance plan, *GIS* ≥ 65 years with guaranteed income supplement, *LRFA* recipients of last resort financial assistance, *COPD* chronic obstructive pulmonary disease, *CHF* congestive heart failure, *CAD* coronary artery disease, *HBP* high blood pressure, *CNCP* chronic non-cancer pain, *CMD* common mental disorders, *SMD* serious mental disorders.

### Main results

Multiple combinations of explicative variables were evaluated. The following variables were selected for their clinical interpretation and explicative power: age, public prescription drug insurance plan status, Charlson comorbidity index, number of previous ED visits, chronic obstructive pulmonary disease, injury, serious mental disorders, common mental disorders, chronic non-cancer pain, alcohol, and drugs. No missing values were observed in the variables selected for prediction.

Model performances are shown in Tables 2, 3, for frequent users 3 and 5 respectively. In both cases, RF1 (binary outcome) had poor performances regarding AUC and SEN, followed by NB (poor or fair). On the other hand, RF1 had the highest SPE and PPV. GBM, LR, NN, and RF2 had similar good performances (or very good in the case of GBM, LR, and RF2 for frequent users 5). Performances improved as the threshold for frequent use was increased from three to five visits, except for RF1. Overall, SPE (NPV) was higher than SEN (PPV).

**Table 2:** Model performances for frequent users 3.

Model	AUC	SEN	SPE	PPV	NPV
LR	74.8 (74.3–75.4)	60.0	78.0	22.3	94.9
GBM	74.9 (74.3–75.5)	64.0	74.0	20.7	95.1
NB	59.6 (59.1–60.0)	23.2	95.9	37.6	92.2
NN	74.4 (73.8–75.0)	58.5	78.8	22.6	94.7
RF 1	53.8 (53.5–54.0)	8.1	99.4	60.2	91.1
RF 2	74.7 (74.1–75.3)	61.8	76.2	21.5	95.0

*GBM* gradient boosting machine, *LR* logistic regression, *NB* naïve bayes, *NN* neural network, *RF* random forests, *AUC* area under the curve, *SEN* sensitivity, *SPE* specificity, *PPV* positive predicted value, *NPV* negative predicted value.

**Table 3:** Model performances for frequent users 5.

Model	AUC	SEN	SPE	PPV	NPV
LR	81.3 (80.4–82.2)	67.7	81.3	10.1	98.8
GBM	81.4 (80.4–82.3)	69.9	78.9	9.3	98.8
NB	61.2 (60.4–62.0)	24.8	97.6	24.5	97.7
NN	78.4 (77.3–79.4)	63.2	82.1	9.9	98.6
RF 1	51.4 (51.1–51.8)	2.9	99.9	51.3	97.1
RF 2	81.1 (80.2–82.0)	72.5	75.6	8.4	98.9

*GBM* gradient boosting machine, *LR* logistic regression, *NB* naïve bayes, *NN* neural network, *RF* random forests (1: binary outcome, 2: continuous outcome). *AUC* area under the curve, *SEN* sensitivity, *SPE* specificity, *PPV* positive predicted value, *NPV* negative predicted value.

Variable importance results are shown in Tables 4, 5. Those measures are relative, meaning that it is only possible to compare importance between variables in the same model (e.g., variable importance between LR and GBM are not comparable). However, the ranking of independent variables in each model can still be compared for all models, along with the relative magnitude. All models reported the number of previous ED visits as the most important variable for prediction. The magnitude by which it was superior to the other variables varied considerably. CCI and PPDIP were also important, but to a lesser extent (for instance, their importance was respectively 6 and 12 times less than PV for RF2 in the case of frequent users 5). Among chronic diseases, COPD was the most important.

**Table 4:** Variable importance for the predictive models (frequent users 5).

Variable	LR	GBM	NN	RF1	RF2
**Age**					
18–34	Ref	Ref	Ref	284.6	134.2
35–54	5.2	6.8E−4	4.3		
55–64	6.2	9.0E−4	4.0		
65–74	6.7	1.4E−3	3.6		
75–84	4.3	6.5E−4	4.7		
≥ 85	2.4	3.4E−3	4.2		
**PPDIP**					
Regular	Ref	Ref	Ref	220.7	105.6
GIS	9.8	1.6E−2	4.9		
Not admissible	5.4	1.1E−2	4.3		
LRFA	11.3	1.8E−2	5.2		
**CCI**					
0	Ref	Ref	Ref	281.4	134.7
1–2	12.9	1.1E−2	3.5		
3–4	13.9	1.4E−2	3.2		
≥ 5	16.4	2.3E−2	4.7		
**PV**					
≤ 1	Ref	Ref	Ref	1845.8	916.7
2–3	49.7	0.1	2.9		
4	54.0	0.1	2.8		
5	53.3	0.1	5.3		
≥ 6	94.8	0.4	4.9		
**COPD**	16.9	0.1	4.3	142.7	70.2
**Injury**	2.9	2.4E−3	4.2	102.2	47.9
**SMD**	4.5	2.4E−3	5.5	93.9	45.1
**CMD**	8.9	3.3E−2	3.9	93.7	45.7
**CNCP**	8.6	5.5E−3	3.7	102.1	47.9
**Alcohol**	5.2	3.5E−3	5.2	90.6	43.2
**Drugs**	6.8	1.3E−2	4.5	123.9	61.4

The higher the value, the higher the importance (relative to a model).

*LR* logistic regression, *GBM* gradient boosting machine, *NN* neural network, *RF* random forests (1: binary outcome, 2: continuous outcome).

**Table 5:** Variable importance for the predictive models (frequent users 3).

Variable	LR	GBM	NN	RF1	RF2
**Age**					
18–34	Ref	Ref	Ref	562.0	271.1
35–54	7.0	5.1E−4	3.5		
55–64	9.0	1.1E−3	2.7		
65–74	7.5	4.4E−4	3.6		
75–84	2.5	4.0E−3	2.8		
≥ 85	3.2	8.5E−3	3.7		
**PPDIP**					
Regular	Ref	Ref	Ref	465.6	226.3
GIS	13.2	7.5E−3	4.2		
Not admissible	8.5	1.1E−2	3.9		
LRFA	17.0	1.4E−2	4.7		
**CCI**					
0	Ref	Ref	Ref	882.1	439.6
1–2	19.8	1.7E−2	4.1		
3–4	21.3	1.3E−2	5.0		
≥ 5	24.7	1.6E−2	4.3		
**PV**					
≤ 1	Ref	Ref	Ref	5255.2	2621.2
2–3	74.3	0.2	3.7		
4	66.2	0.1	5.3		
5	61.7	0.1	6.0		
≥ 6	96.4	0.3	5.3		
**COPD**	24.4	2.6E−2	5.1	475.9	234.7
**Injury**	5.1	2.2E−3	3.7	205.3	98.3
**SMD**	5.4	2.2E−3	4.8	182.0	88.5
**CMD**	12.0	1.8E−2	2.9	266.9	130.8
**CNCP**	11.6	5.7E−3	3.3	180.1	86.7
**Alcohol**	4.3	2.0E−3	5.3	164.6	79.7
**Drugs**	8.1	2.9E−3	4.8	228.4	111.2

The higher the value, the higher the importance (relative to a model).

*LR* logistic regression, *GBM* gradient boosting machine, *NN* neural network, *RF* random forests (1: binary outcome, 2: continuous outcome).

No significant changes were observed in the interpretation of results during sensitivity analyses.

## Limitations

Both quantity and quality of data are imperative in a ML context. In this study, we had access to an exhaustive medico-administrative database which included hospital and physician data, but it did not include patient reported outcomes (e.g., perceived health, included in the Canadian Community Health Survey^46^). Those latter could improve the predictive power of models in future work. For instance, studies using national health surveys and telephone interviews found that fair or poor health status and dissatisfaction with treatment outcome were significantly associated with frequent ED use^47,48^.

Our study focused on frequent ED users with chronic diseases; though results should only be generalized to this population, chronic diseases are common in the frequent ED user population. Better understanding of a population of ED users with chronic diseases is relevant for other healthcare aspects as chronic diseases are linked not only to frequent ED use, but also to hospitalisations, functioning, and deaths^24,49,50^.

## Discussion

This paper aimed at comparing four ML prediction models (gradient boosting machine, naïve Bayes, neural networks, and random forests) with logistic regression, for frequent ED use in a population with chronic diseases. Those ML models have been successfully used to predict related issues, such as ED revisits, in hospital mortality, or hospital admissions at ED triage^42,51,52^. Accurate ML models may help for early identification of frequent ED use, thus improving targeted interventions such as case management^2,15,16^. To this end, case-finding tools are appropriate, such as CONECT-6 which was derived from LR models^53^.

### Model performance

In our study, no model clearly outperformed the others. Other studies on frequent ED use that applied ML reached a similar conclusion^18,20,54^, though they either focused on a specific chronic disease such as asthma or epilepsy or used hospital only data. In fact, a recent systematic review aiming at comparing performances of LR with ML models (among which figured the ones used in this study) for clinical prediction of a binary outcome showed that there is currently no clear performance benefit of ML models^55^. However, this review included only studies that used clinical data. Other studies that focus on ED related issues (e.g. risk of emergency hospital admission, risk for sepsis, heart failure readmission) found improved predictions with ML^56,57^, although this is not a general rule^58^. Quantity of variables (58 to 121 variables^56^) or very discriminative variables^57^ explained those improved predictions, amongst others. In our models, increasing the threshold for frequent ED use (thus reducing the number of frequent ED users) gave slightly better performances for all models. A higher threshold increased the homogeneity of the characteristics of frequent users, thus facilitating prediction of their ED use, a result that has already been observed^54^. Other studies also compared LR to ML models using administrative claims data^59,60^ and found similar performances, though they did not focus on ED-related outcomes.

In medical studies, the signal-to-noise ratio is often low, i.e., the amount of information contained in the database that is useful for the prediction^61^, which may explain in part the modest improvements (if any) of ML models. The type of available variables may also affect performance. For instance, in a study about uncontrolled diabetes prediction, LR was outperformed by NN or GBM^62^. The authors used data from administrative claims and from US census, in which they had access to social determinants, such as food insecurity or recreational park access. It is possible to tune more precisely ML models to overcome those limitations. In our study, this fine-tuning would have amounted to evaluate model parameters over broader spaces. As an example, NN is known for its ability to model complex and nonlinear relationships by combining multiple hidden layers^41^, which is limited for a traditional LR. Broader ranges could also be evaluated; GBM has shown good performances and helped refine clinical tools when allowed to learn slowly^57^. However, this fine-tuning comes with a high computation cost and an added complexity. This latter drawback may result in overfitting issues and limited generalization.

Our models had higher sensitivity than positive predictive value, apart from RF1. This means that most models accounted for a fair portion of frequent ED users, but the number of false positives was significant. This contrasts with another study on frequent ED use among children with asthma^20^. The authors also applied ML models (LR and RF amongst others) and found higher predictive positive values^48–70^ than sensitivity^16–27^. However, their threshold choice was guided by a maximization of the AUC rather than by a statistical criterion. Besides, specificity and negative predicted values were high in our study. This is a known issue when dealing with imbalanced health datasets (frequent users 3 and 5 represented 9.5% and 3.0% of the cohort respectively)^63,64^. Algorithms learns mostly from the majority class, which introduces a bias towards non-frequent users. There are possible adjustments such as under-sampling from the majority class or over-sampling from the minority class, but those may not be recommended procedures as they distort prevalence^55^. Learning from highly imbalanced datasets is an active research area^65^ and may affect prediction for frequent ED use in the future, especially if combined with multiple different models^66^.

### Variable importance

The models developed in our study are also interesting from a clinical point of view (i.e., risk stratification by variable for frequent ED use). In our study, CCI, PPDIP, and the previous number of ED visits were important, though the latter was the most important variable by a large margin. This result is supported by other studies on frequent ED use conducted with LR^26,67,68^, but also with ML models^18,20^. In fact, this variable is usually so important that Brennan et al.^68^ stated that “targeting patients with the most extreme number of ED visits may be the best and most practical option for targeted interventions”, thus allowing for optimal resource allocation. Hudon *et al*. (2020) also found that a LR including this variable and having a previous hospitalization performed almost as well as models with more variables such as comorbidities, sociodemographic status, and public prescription drug insurance status^26^. Even when predicting other ED related-outcomes, the previous number of visits is relevant: Rahimian et al.^56^ predicted emergency hospital admission (after an ED visit) using RF and GBM and found that it was the most important variable. They also found that other variables are excellent predictors of emergency hospital admission, such as laboratory test results (e.g., cholesterol ratio, haemoglobin, platelets).

## Conclusions

Frequent ED use is a major issue in primary and emergency care, and ML models are becoming increasingly popular in medicine and healthcare in general. They are rapidly evolving, offering new opportunities, and while there has been substantial theoretical progress with ML models, the small improvements do not show a clear superiority over simpler models. Those latter still display reasonable performances^55,69^. In our study, LR was as successful in predicting frequent ED use as other ML models, while the number of ED visits was the most important variable. Access to other variables may be more helpful for refining prediction in the case of frequent ED use, such as patient-reported outcomes or clinical notes. Those types of data have been successfully used with machine learning models in a context of primary care, although not for ED use prediction^70,71^. Future work also includes considering complex non-linear interactions, where ML models outperform traditional ones^72^.

## Supplementary Information

Supplementary Information.

## Data Availability

The datasets analysed during the current study are not publicly available due to provincial laws about privacy. Although we acknowledge the importance of data availability for reproducible research, our research team is bound by legal reasons to not divulge any part of the data. The *Commission de l’accès à l’information du Québec* is the provincial organisation that reviews research projects and allows researchers to access health databases. It is also responsible for ensuring their privacy as those databases contain sensitive patient information and it does not legally allow for making any part of them public. Therefore, we are not able to make any part of our data publicly available.

## Abbreviations

AUC: Area under the curve
CAD: Coronary artery disease
CCI: Charlson comorbidity index
CHF: Congestive heart failure
CMD: Common mental disorders
CNCP: Chronic noncancer pain
COPD: Chronic obstructive pulmonary disorder
ED: Emergency department
HBP: High blood pressure
GBM: Gradient boosting machine
LR: Logistic regression
ML: Machine learning
NB: Naïve bayes
NN: Neural network
NPV: Negative predicted value
PPDIP: Public prescription drug insurance plan
RF: Random forests
SEN: Sensitivity
SMD: Serious mental disorders
SPE: Specificity
PPV: Positive predicted value
